# Commentary: A tribute to Dr. Paul Berg (1926–2023) American biochemist, Nobel Laureate and discoverer of recombinant DNA technology, vaccine and genetic engineering

**DOI:** 10.3389/fcell.2023.1210530

**Published:** 2023-05-18

**Authors:** Walter J. Lukiw

**Affiliations:** LSU Health Sciences Center New Orleans, Louisiana State University, New Orleans, LA, United States

**Keywords:** recombinant DNA technology, SV40, Paul Berg, Frederick Sanger, Walter Gilbert, Asilomar conference on recombinant DNA, Nobel Prize in Chemistry 1980, recombinant DNA

The world of human molecular genetics, virology and immunology has lost one of the pillars in the study of recombinant DNA technology and vaccine and genetic engineering with the passing of Paul Berg. Inventor of one of the most powerful and trusted tools in the armamentarium of modern molecular biology, Paul Berg was the first biochemist-geneticist to incorporate DNA from the bacterium *Escherichia coli* into the potentially cancer-causing DNA polyoma tumor virus SV40, as was reported by his laboratory in late 1972 in the now classic paper in the Proceedings of the National Academy of Science United States (Jackson et al., 1972). Berg thereby created the first DNA molecule made from different parts of the genome of different organisms. This type of molecule became known as “hybrid DNA” or “recombinant DNA” with the idea to use the modified SV40 as a viral vector to carry “foreign” or “intelligent and strategically designed” genetic information into animal cells. Berg also discovered, and in part characterized transfer RNA (tRNA) and showed that amino acids needed to be activated before being attached to tRNA and before their assembly into proteins. Berg also was the first to purify the enzyme that carried out the formation of acetyl CoA, made deep inroads into our understanding of “activated acetate” and mechanisms related to fatty acid activation and polymerization and was the first to characterize the distribution of CpG dinucleotides in the human genome that distinguishes two distinct classes of eukaryotic gene promoters (Saxonov et al., 2006). Berg’s novel recombinant DNA discoveries are the basis for the biotechnology industry today worth hundreds of billions of dollars, that involve technologies capable of the editing of heritable genes in humans and the genetic engineering of new therapeutic treatments for many diseases and of vaccines, like the messenger RNA (mRNA)-based therapeutics currently used to counter the SARS-CoV-2 virus, the causative agent of COVID-19.

Born in New York in 1926 Paul Berg obtained a doctoral (PhD) degree in biochemistry from Case Western Reserve University OH, United States, and carried out postdoctoral studies with Herman Moritz Kalckar (1908–1991) in Copenhagen, Denmark, and Arthur Kornberg (1918–2007) at the National Institutes of Health, Bethesda MA, United States and at Washington University Missouri MO, United States. Berg joined the Department of Biochemistry at Stanford University CA, United States in late 1959 where he spent the remainder of his professional career. He served as director of the Beckman Center for Molecular and Genetic Medicine at Stanford for 15 years. Berg was awarded a half-share of the 1980 Nobel Prize in Chemistry “for his fundamental studies in the biochemistry of nucleic acids, with particular regard to recombinant DNA technology” which he shared with fellow biochemist Frederick Sanger (1918–2013) then at the Medical Research Council Laboratory of Molecular Biology in Cambridge, United Kingdom and the molecular biologist Walter Gilbert (1932-present) then at Harvard University in Boston MA, United States “for their contributions concerning the determination of base sequences in nucleic acids” (Nobelprize, 2023).

An extraordinary scientific researcher, teacher, lecturer, mentor, advisor and administrator, Berg was a capable and accomplished proponent of advocating common sense approaches to contentious and controversial public-, medical- and scientific-policy issues, including the conscientious and responsible use of recombinant DNA and embryonic stem cell technologies in human genetic, molecular biological and biomedical investigation (Med.stanford, 2022). Indeed Berg will be remembered for devoting a considerable amount of time to promoting the safe and sensible use of recombinant DNA, stem cell and related DNA-based technologies. Soon after his DNA recombination discoveries, concerns arose that recombining the genes of SV40 and *E. coli* and other common human gastrointestinal (GI) tract bacteria could raise the risk of human cancers, prompting Berg to circulate what became known as the “Berg Letter” to several prominent journals, including *Nature*, requesting that members of the scientific community call a moratorium on all recombinant DNA work. Organized by Paul Berg, the Asilomar Conference on Recombinant DNA held in Pacific Grove CA, United States in 1975 set the first standards that allowed geneticists to advance genetic research to its limits without endangering public health (Berg, 2008).

Together with Arthur Kornberg and US geneticist Charles Yanofsky in 1980 Berg, was the cofounder of the DNAX Research Institute of Cellular and Molecular Biology in Palo Alto CA, United States, a biotechnology research institute focusing on the ethical use and implementation of recombinant DNA technologies related to nucleic acid biochemistry and the advancement of vaccine and genetic engineering to extend the medical and moral health, bioethics and the welfare and prosperity of all of humanity.
